# Microbiological and Clinical Characteristics of Bloodstream Infections in General Intensive Care Unit: A Retrospective Study

**DOI:** 10.3389/fmed.2022.876207

**Published:** 2022-04-28

**Authors:** He-Ning Wu, Er-Yan Yuan, Wen-Bin Li, Min Peng, Qing-Yu Zhang, Ke-liang Xie

**Affiliations:** ^1^Department of Critical Care Medicine, Tianjin Medical University General Hospital, Tianjin, China; ^2^Department of Geriatrics, Tianjin Medical University General Hospital, Tianjin, China; ^3^Department of Gastroenterology, Tianjin Medical University General Hospital, Tianjin, China; ^4^Department of Anesthesiology, Tianjin Institute of Anesthesiology, Tianjin Medical University General Hospital, Tianjin, China

**Keywords:** bloodstream infection, antibiotic resistance, procalcitonin, multidrug-resistance, vancomycin-resistant enterococci (VRE)

## Abstract

**Background:**

Bloodstream infections (BSI) are one of the common causes of morbidity and mortality in hospitals; however, the pathogenic spectrum and bacterial antibiotic resistance vary across the world. Therefore, identifying the pathogenic spectrum and changes in bacterial antibiotic resistance is critical in controlling BSI and preventing the irrational use of antibiotics. This study evaluated the microbiological and clinical data of BSI patients in the intensive care unit (ICU) of Tianjin Medical University General Hospital in Tianjin, China, to guide the selection of empirical antibiotic therapy.

**Methods:**

This study retrospectively analyzed the distribution and antibiotic resistance of pathogens based on the clinical data of BSI patients presented in the ICU of a tertiary teaching hospital from 2018 to 2020. Test performance for the prediction of pathogen species was assessed by receiver operating characteristic (ROC) analysis.

**Results:**

The analysis of the data of 382 BSI cases (10.40 cases per thousand patient day) revealed the most frequently isolated microorganisms to be *Klebsiella pneumonia* (11.52%), followed by *Escherichia coli* (9.95%), *Staphylococcus epidermidis* (9.95%), *Candida parapsilosis* (8.12%), and *Enterococcus faecium* (8.12%). Out of the isolated *E. coli* and *K. pneumonia* strains, 52.63, and 36.36%, respectively, were extended-spectrum β-lactamase (ESBL) positive. The antibiotic-resistance rate of the ESBL-positive strains was 30.56% for piperacillin/tazobactam, 5.56% for imipenem, and 11.11% for tigecycline. In addition, most *A. baumannii* belonged to the group of multidrug-resistant (MDR) strains, with an antibiotic-resistance rate of 90.48% for meropenem and 16.00% for amikacin. However, polymyxin-resistant *A. baumannii* strains were not detected. Four strains of methicillin-resistant *S. aureus* (MRSA) (4/21, 19.05%) and one strain of vancomycin-resistant enterococci (VRE) were detected, with a resistance rate of 4.76 and 2.32%, respectively. Among the isolated 55 fungal strains, *C. parapsilosis* was the most common one (30/55, 56.36%), with an antibiotic-resistance rate of 5.77% for voriconazole, fluconazole, and itraconazole. The presence of amphotericin B-or flucytosine-resistant strains was not observed. Compared with the patients with Gram-positive and fungal pathogens, patients with Gram-negative bacteria exhibited the highest sequential organ failure assessment (SOFA) score (*P* < 0.001), lowest Glasgow Coma Scale (GCS) (*P* = 0.010), lowest platelet (PLT) value (*P* < 0.001), highest plasma creatinine (Cr) value (*P* = 0.016), and the highest procalcitonin (PCT) value (*P* < 0.001). The AUC in the ROC curve was 0.698 for the differentiation of Gram-negative BSI from Gram-positive BSI. A cutoff value of 8.47 ng/mL for PCT indicated a sensitivity of 56.9% and a specificity of 75.5%. The AUC in the ROC curve was 0.612 for the differentiation of bacteremia from fungemia. A cutoff value of 4.19 ng/mL for PCT indicated a sensitivity of 56.8% and a specificity of 62.7%.

**Conclusion:**

Among the bloodstream infection strains in ICU, Gram-negative bacteria have the highest drug resistance rate, and will cause more serious brain damage, renal function damage and thrombocytopenia. So clinician should pay more attention to the treatment of Gram-negative bacteria in patients with bloodstream infection in ICU. The test index of PCT can be used to distinguish Gram-negative bacteremia from Gram-positive and bacteremia from fungemia but not as an effective indicator, thereby indicating the need for further large-scale research.

## Introduction

Bloodstream infections (BSI) are a life-threatening condition affecting patients in intensive care units (ICUs). The timely and effective application of antibiotics is crucial for managing the morbidity and mortality of the infection (1, 2). Antibiotics are useful for infection control, but their overuse or misuse could induce antibiotic resistance in various pathogens (3–5). For example, penicillin resistance was first reported 80 years ago (6). Currently, antibiotics and antibiotic-resistance genes have been reported in surface water, effluents from sewage treatment plants, soils, and animal wastes (7). Owing to this wide distribution, WHO declared it as a serious public health crisis of the 21st century (8). Generally, the results of etiological tests become available after 3–5 days only. Therefore, before obtaining the etiological results, most clinicians follow the empirical anti-infective therapy for the choice of the antibiotic regimen. The international Surviving Sepsis Campaign (SSC) recommended the empirical broad-spectrum therapy to be initiated immediately, with one or more intravenous antimicrobials to cover all likely pathogens (9). Thus, the use of broad-spectrum antibiotics is closely related to the emergence of bacterial resistance. All these factors lead to ICU being not only the ward with the highest use of broad-spectrum antibiotics but also the high-risk area of antibiotic-resistant bacterial infections.

Owing to increasing bacterial resistance, the choice of empirical antibiotics has become the focus of clinical treatments. In this research, we analyzed the clinical data and bacterial antibiotic resistance of BSI patients presented in ICU from January 2018 to December 2020. In addition, we explored the relationship between the changes in clinical data and bloodstream infection agents to guide the selection of empirical antibiotic therapy.

According to the National Antimicrobial Stewardship Campaign in China, to control the irrational consumption of antibiotics, especially the prediction usage in surgery, all antibiotic prescriptions should follow the drug use list (10).

## Materials and Methods

This retrospective study was conducted in Tianjin Medical University General Hospital in Tianjin, China, from January 2018 to December 2020 and was approved by the hospital’s ethics committee (NO. IRB2021-YX-062–01). This tertiary teaching hospital is a 2,468-bed facility, and the General Intensive Care Unit (GICU) comprises 42 beds. All patients over 18 years of age with a confirmed BSI during ICU admission were included in this study. The ICU-acquired BSI was defined as bacteremia or fungemia diagnosed from day 3 onward of ICU stay, with the initial day of ICU admission being designated as day 0.

Blood culture is the golden criterion for the diagnosis of BSI. In our department, blood culture was performed for patients with infectious symptoms, such as fever, cold, shivering, and low blood pressure, before administering antimicrobial therapy. The specimens were sent to the microbiology laboratory, and the VITEK-2 compact automated system was used for bacterial identification and antibiotic susceptibility testing. The ATB-Fungus 3 system was used for antifungal susceptibility testing. The antimicrobial susceptibility tests were interpreted according to the guidelines of the Clinical and Laboratory Standards Institute (CLSI). For results not included in CLSI, the guidelines prescribed by the European Committee on Antimicrobial Susceptibility Testing (EUCAST) were referred. Our hospital has developed a critical value reporting system, according to which, if the blood culture results are positive, the microbiology laboratory must report Gram-positive, Gram-negative, or candida as soon as possible; the rapid reporting could direct the clinician’s decision and reduce the delay in initiating the antibiotic treatment. However, generally, it takes 3–5 days to get the final results.

The clinician explained the blood culture results according to the clinical symptoms, treatment effects, bacterial species (e.g., coagulase-negative Staphylococci, Corynebacterium species, *Propionibacterium acnes*, and other skin colonizers), etc., to find contaminants. The bacterial contaminants were recorded in the clinical course record. The clinical course records were reviewed in this study, and the contaminant cases were excluded.

The patients’ data, including gender, age, diagnosis, Acute Physiology, and Chronic Health Evaluation II score (APACHE II), Sequential Organ Failure Assessment (SOFA) score, Glasgow Coma Scale (GCS), mechanical ventilation, catheter insertion, hemofiltration, prescription, vital signs, vasopressors, length of stay in ICU, antibiotics consumption, and prognosis were extracted from the health information system (HIS). The laboratory information system (LIS) provided the patients’ examination results, such as microorganisms isolated from samples, resistance to antimicrobials, blood routine, blood creatine, alanine aminotransferase (ALT), aspartate aminotransferase (AST), total bilirubin (TBIL), PCT, and arterial blood gas analysis. All examination results were submitted within 24 h after blood culture. APACHE II score, SOFA score and GCS score were calculated within 24 h after blood culture.

**FIGURE 1:**
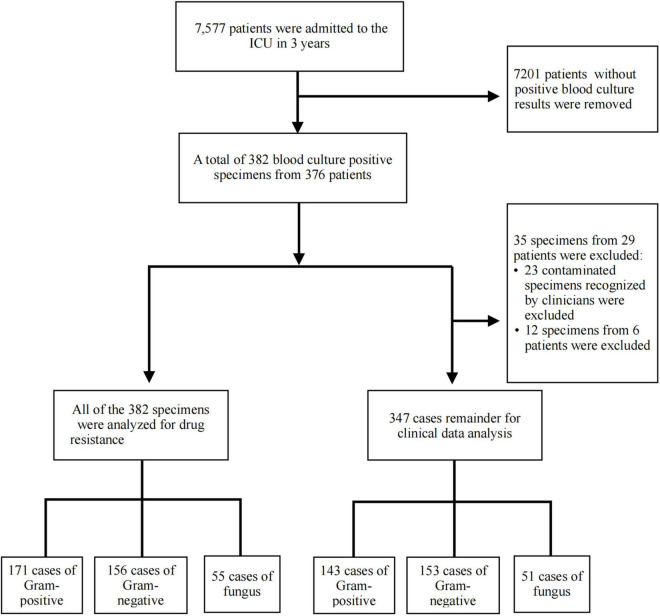
Study flow diagram. A total of 382 blood culture positive specimens were analyzed for drug resistance, including 171 cases of Gram-positive bacteria, 156 cases of Gram-negative bacteria, and 55 cases of fungus. After excluding contaminated strains recognized by clinicians and one bottle with more than one organism, 347 cases of bloodstream infection (BSI) remainder were included in the clinical data analysis, including 143 cases of Gram-positive bacteria, 153 cases of Gram-negative bacteria, and 51 cases of fungus.

According to the blood culture results, we divided the patients into the following three groups: Gram-positive bacteremia, Gram-negative bacteremia, and fungemia. The collected etiological results were analyzed for drug resistance. To exclude the influence of simultaneous infection of two or more pathogens on patients’ clinical symptoms and laboratory indexes, the data of cases with two or more pathogens in one culture medium were excluded from this study (Figure 1).

## Data Analysis

The WHONET 5.6 software was used to evaluate the blood culture results and the antimicrobial-resistance trends.

Statistical analyses were performed using the SPSS 24.0 software. Categorical data were described as percentages. Continuous variables were given as mean [standard deviation (SD)] for normal data and median [interquartile range (IQR)] for non-normal data. The differences between the observed and expected frequencies were calculated using the Chi-square test for the categorical variables, whereas the *T*-test and ANOVA were used to compare the continuous variables. The Wilcoxon rank sum test is applied to the count data of non-normal distribution. The value of *P* < 0.05 was considered as statistically significant.

Receiver operating characteristic (ROC) curves were used to determine the predictive performance of procalcitonin (PCT) for pathogen species. MedCalc software was used for ROC analysis.

## Results

### General Situation

From January 2018 to December 2020, the total number of patients admitted to the ICU was 7,577 (36,716 patient day), including 2,398 (12,119 patient day) in 2018, 2,455 (12,158 patient day) in 2019, and 2,724 (12,439 patient day) in 2020. Furthermore, the mortality rate was 4.14% (99/2398) in 2018, 3.75% in 2019 (92/2455), and 2.06% in 2020 (56/2724). In this study, a total of 382 BSI cases (10.40/1,000 patient day) were included, with 171 cases of Gram-positive bacterial infections (44.76%), 156 cases of Gram-negative bacterial infections (40.84%), and 55 cases of fungal infections (14.40%). Out of the 382 BSI cases, cases with contaminated bacteria and more than one organism were excluded, leading to 347 BSI cases, as depicted in Figure 1. The analysis of the 347 cases revealed 143 Gram-positive bacterial infections, with a mortality rate of 30.77% (44/143); 153 Gram-negative bacterial infections, with a mortality rate of 33.99% (52/153); and 51 fungal infections, with a mortality rate of 39.22% (20/51). The year-wise analysis indicated 93 BSI cases in 2018, with a mortality rate of 40.86% (38/93); 120 BSI cases in 2019, with a mortality rate of 35.00% (42/120); and 134 BSI cases in 2020, with a mortality rate of 27.61% (37/134).

### Clinical Data Analysis

The remaining patients ’ data were analyzed except for the cases with two or more strains in the same culture medium (Table 1).

**TABLE 1:** The characteristics of the included patients, except for one case with more than one strain.

Characteristics	G^+^ (143)	G^–^ (153)	Fungi (51)	F/χ ^2^ Value	*P*-value
Male (%)	93 (65.035)	86 (56.209)	28 (54.902)	2.953^b^	0.228
Age mean ± SD	61.22 ± 16.835	62.13 ± 15.396	66.24 ± 14.685	1.894^a^	0.152
Community acquired infection (*n*, %)	46 (32.168)	57 (37.255)	9 (17.647)	6.729^b^	**0.035**
Hospital acquired infection (except ICU) (*n*, %)	23 (16.084)	27 (16.647)	8 (15.686)	0.175^b^	0.916
ICU acquired infection (*n*, %)	74 (51.748)	69 (45.098)	34 (66.667)	7.174^b^	**0.028**
History of hormone or immunosuppressant use (*n*, %)	21 (14.69)	31 (20.26)	5 (9.80)	3.584^b^	0.167
Elective surgery (*n*, %)	43 (30.70)	52 (33.99)	14 (27.45)	0.889^b^	0.641
Emergency surgery (*n*, %)	24 (16.78)	28 (18.30)	16 (10.46)	5.251^b^	0.072**
Mechanical ventilation (hour)	333.69 (0–4729)	312.70 (0–2885)	521.882 (0–6254)	1.722^a^	0.180
Catheter insertion					
Internal jugular vein catheterization (*n*, %)	87 (60.84)	91 (59.48)	31 (60.78)	0.065^b^	0.968
Subclavian vein catheterization (*n*, %)	18 (12.59)	17 (11.11)	9 (17.65)	1.478^b^	0.478
Femoral vein catheterization (*n*, %)	57 (30.89)	60 (39.22)	21 (41.18)	0.013^b^	0.993
ICU length of stay (days) (median, IQR)	30.50 (1–261)	25.14 (1–249)	44.25 (1–865)	2.205^a^	0.112
Apache II score (median, IQR)	21.58 (7–41)	23.14 (8–45)	21.69 (10–41)	1.662^a^	0.191
SOFA score (median, IQR)	7.54 (1–20)	9.36 (0–20)	6.43 (0–18)	9.712^a^	**< 0.001**
GCS score (median, IQR)	6.61 (3–15)	5.93 (3–15)	8.92 (3–15)	4.719^a^	**0.010**
Clinical data					
WBC (*10^9^/L) mean ± SD	14.396 ± 9.735	15.174 ± 10.965	13.554 ± 7.438	0.559^a^	0.573
PLT (*10^9^/L) (median, IQR)	140.06 (6–521)	97.52 (1–469)	137.04 (7–339)	8.565^a^	**< 0.001**
PCT (ng/mL) (median, IQR)	14.52 (0.021–243.33)	42.618 (0.05–298.01)	12.14 (0.13–110.93)	13.522^a^	**< 0.001**
Cr (μmol/L) (median, IQR)	168.82 (15–768)	222.45 (17–1430)	153.80 (17–523)	4.208^a^	**0.016**
ALT (U/L) (median, IQR)	114.03 (4–2928)	214.10 (6–3480)	148.49 (6–1981)	2.461^a^	0.087*
AST (U/L) (median, IQR)	168.24 (4–3004)	316.59 (12–7992)	230.35 (9–4311)	1.666^a^	0.191*
TBIL (μmol/L) (median, IQR)	48.30 (4.9–399.0)	67.86 (3.5–563.5)	53.99 (5.0–366.4)	2.109^a^	0.123*
Vasopressors (*n*, %)	49 (34.27)	70 (45.75)	14 (27.45)	7.118^b^	**0.028**
Mean arterial pressure (mmHg) (median, IQR)	80.9 (42–161)	75.59 (31–131)	81.78 (44–128)	2.910^a^	0.056*
Lactate (mmol/L) mean ± SD	3.363 ± 2.665	3.821 ± 3.624	2.600 ± 2.666	3.015^a^	0.050**
A-aDO_2_ (mmHg) (x ± 95% CI)	238.415 (215.414, 261.416)	258.866 (234.729, 283.002)	234.586 (198.411, 270.762)	1.561^b^	0.458

G^+^: gram positive; G^–^: gram negative; F: ANOVA test; χ^2^: chi-square test; p-value: <0.05 was considered as statistically significant. ^a^for F, ^b^for χ^2^;* for p < 0.05, comparison between Gram-negative BSI and Gram-positive BSI; ** for p < 0.05, comparison between bacteremia and fungemia.

Results indicated that Gram-negative bacteria were the most common strains in the community-acquired BSI cases (57/112, 50.89%), Gram-positive bacteria were the most common strains in the ICU-acquired BSI cases (74/177, 41.81%), and fungemia mainly occurred in the ICU-acquired BSI patients (34/51, 66.67%). Gram-negative bacteria were the most pathogenic bacteria in patients treated with hormones or immunosuppressants (31/57, 54.39%, *P* = 0.167); however, the difference was not statistically significant. The duration of ICU hospitalization (44.25 days, *P* = 0.112) and mechanical ventilation (521.882 h, *P* = 0.180) of patients with fungemia were longer than the other two groups, without any statistically significant difference.

Acute Physiology, and Chronic Health Evaluation II score and SOFA score are widely used in ICUs to evaluate the severity of the disease, the higher the score, the higher the severity of the disease. The average score of APACHE II was greater than 20 in the three groups, higher than the critical condition standard of 15. The highest SOFA score was obtained for the Gram-negative group (*P* < 0.001). In the Gram-negative group, an increased number of patients used vasopressor (*P* = 0.028) and exhibited the lowest mean blood pressure (*P* = 0.056) and highest arterial blood lactate levels (*P* = 0.050). However, the differences for mean blood pressure and arterial blood lactate levels were not statistically significant. The GCS score can objectively reflect patient’s coma severity. The Gram-negative group exhibited the lowest GCS score (*P* = 0.010), which suggested the most serious degree of brain injury in this group. In addition, the Gram-negative group exhibited lowest PLT value (*P* < 0.001), highest plasma CR value (*P* = 0.016), and highest PCT value (*P* < 0.001). However, no differences in white blood cell (WBC) count (*P* = 0.573) were observed among the three groups. Subgroup comparison showed Gram-negative group patients with lower mean blood pressure (*P* = 0.035) and higher ALT (*P* = 0.010), AST (*P* = 0.039), and TBIL (*P* = 0.025) values compared with Gram-positive group patients. Compared with fungemia patients, bacteremia patients showed higher arterial lactic acid level (*P* = 0.001).

**FIGURE 2:**
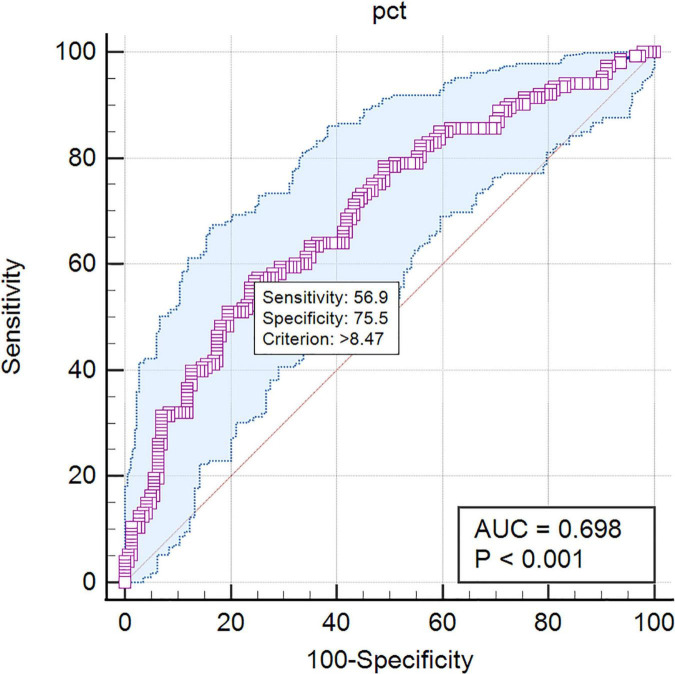
The ROC curves of PCT predicting Gram-negative bacteremia from Gram-positive bacteremia. PCT: AUC = 0.698, cutoff point 8.47 ng/mL, sensitivity 56.9%, specificity 75.5%.

**FIGURE 3:**
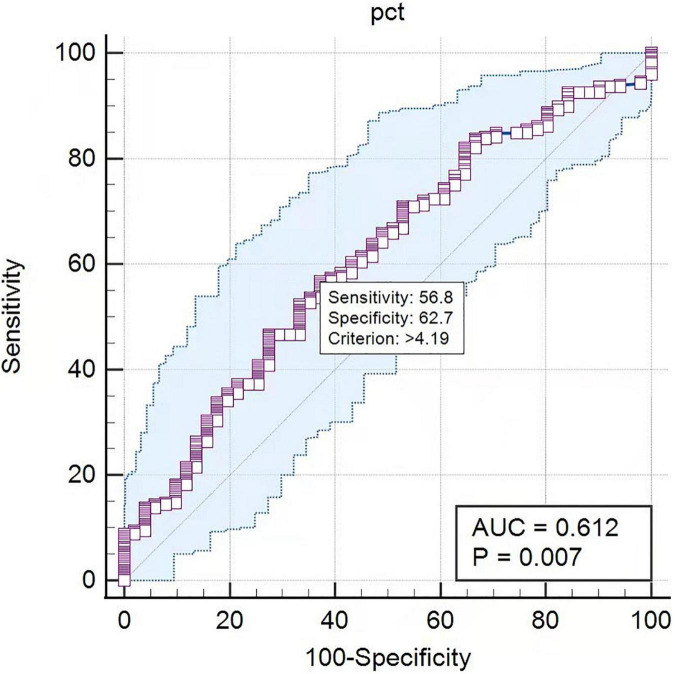
The ROC curves of PCT predicting bacteremia from fungemia. PCT: AUC = 0.612, cutoff point 4.19 ng/mL, sensitivity 56.8%, specificity 62.7%.

Procalcitonin is a marker of infection correlated with the severity of microbial invasion. In this study, PCT levels were calculated to differentiate pathogen species. The AUC in the ROC curve was 0.698 for the differentiation of Gram-negative BSI from Gram-positive BSI. A cutoff value of 8.47 ng/mL for PCT indicated a sensitivity of 56.9% and a specificity of 75.5% (Figure 2). The AUC in the ROC curve was 0.612 for the differentiation of bacterial BSI from fungal BSI. A cutoff value of 4.19 ng/mL for PCT indicated a sensitivity of 56.8% and a specificity of 62.7% (Figure 3). In the subgroup analysis, no differences in PCT concentrations were observed among the patients infected with *E. coli*, *K. pneumonia*, *P. aeruginosa*, and *A. Baumannii* (*P* = 0.200). In addition, in blood culture, *E. coli* and *P. aeruginosa* were associated with two or three times higher PCT values than *K. pneumonia* and *A. baumannii* (Table 2).

**TABLE 2:** PCT values associated with different pathogens.

Pathogen species detected from blood cultures	Number	PCT (IQR)	*P*-value
*E. coli*	38	66.272 (37.325, 95.218)	0.200
*K. pneumonia*	44	29.170 (16.180, 42.160)	
*P. aeruginosa*	16	69.706 (17.537, 121.874)	
*A. baumannii*	25	19.460 (6.251, 32.668)	

### Bacteriological Results

**FIGURE 4:**
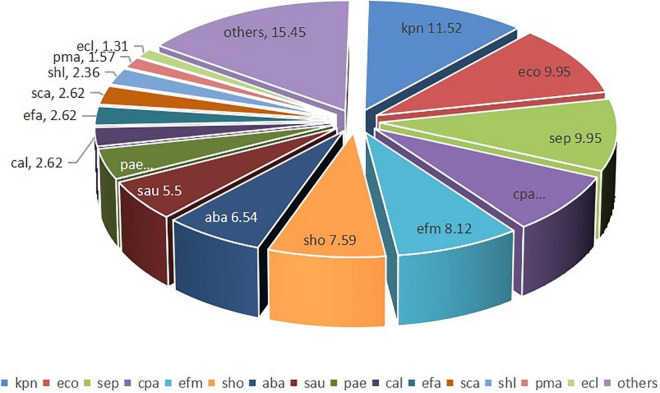
Distribution of the isolated pathogens in blood culture samples (%). kpn, *K. pneumonia*; eco, *E. coli*; sep, *S. epidermidis*; cpa, *C. parapsilosis*; efm, *E. faecium*; sho, *S. hominis*; aba, *A. baumannii*; sau, *S. aureus*; pae, *P. aeruginosa*; cal, *C. albicans*; efa, *E. faecalis*; sca, *S. capitis*; shl, *S. haemolyticus*; pma, *Stenotrophomonas maltophilia*; and ecl, *E. cloacae.*

The most frequently isolated microorganisms were *K. pneumonia* (44/382, 11.52%), *E. coli* (38/382, 9.95%), *S. epidermidis* (38/382, 9.95%), *C. parapsilosis* (31/382, 8.12%), *E. faecium* (31/382, 8.12%), *S. hominis* (29/382, 7.59%), *A. baumannii* (25/382, 6.54%), *S. aureus* (21/382, 5.50%), *P. aeruginosa* (16/382, 4.19%), and *C. albicans* (10/382, 2.62%) (Figure 4).

#### Gram-Negative Bacteria

**FIGURE 5:**
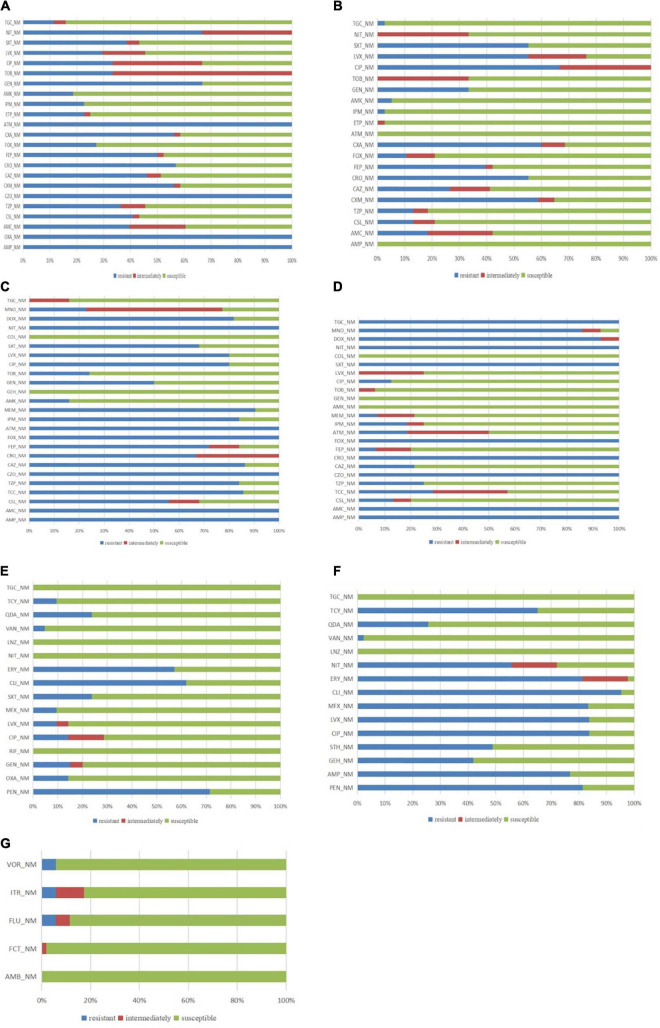
Antibiotic sensitivity patterns of panel **(A)** *K. pneumonia*; **(B)** *E. coli*; **(C)** *A. baumannii*; **(D)** *P. aeruginosa*; **(E)** *S. aureus*; **(F)** Enterococcus; and **(G)** fungal strains; AMB: Amphotericin B; AMC: Amoxicillin + Clavulanate; AMK: Amikacin; AMP: Ampicillin; ATM: Aztreonam; CAZ: Ceftazidime; CIP: Ciprofloxacin; CLI: Clindamycin; COL: Colistin; CRO: Ceftriaxone; CSL: Cefoperazone + Sulbactam; CXM: Cefuroxime; CZO: Cefazolin; DOX: Doxycycline; ERY: Erythromycin; FEP: Cefepime; FCT: Fluorocytosine; FLU: Fluconazole; FOX: Cefoxitin; GEN: Gentamicin; GEH: High concentration gentamicin; IPM: Imipenem; ITR: Itraconazole; LNZ: Linezolid; LVX: Levofloxacin; MEM: Meropenem; MFX: Moxifloxacin; MNO: Minocycline; NIT: Nitrofurantoin; OXA: Oxacillin; PEN: Penicillin; QDA: Quinuptin/dafoptin; RIF: Rifampicin; STH: High concentration streptomycin; SXT: Trimethoprim + Sulfamethoxazole; TCC: Ticarcillin + Clavulanate; TCY: Tetracycline; TGC: Tigecycline; TOB: Tobramycin; TZP: Piperacillin + Tazobactam; VAN: Vancomycin; and VOR: Voriconazole.

The most commonly isolated Gram-negative bacteria were *K. pneumonia.* Out of the 44 isolated strains, 16 were extended-spectrum β-lactamase (ESBL) positive (16/44, 36.37%). This group of bacteria exhibited an antibiotic-resistance rate of approximately 50% for cefuroxime, ceftazidime, ceftriaxone, and cefepime and 22.73% (10/44) for carbapenems. Carbapenemase assay was not performed during the period of this study in our hospital (Figure 5A). The resistance rate for β-lactase inhibitor combinations was in the range of 36.37–40.91%.

The second commonly isolated Gram-negative bacteria was *E. coli*, with 38 strains detected; out of these, 20 cases were ESBL positive (20/38, 52.63%). This group exhibited antibiotic-resistance rates, with 58.82% for cefuroxime, 52.26% for ceftriaxone, 55.26% for quinolones, and 66.67% for ciprofloxacin. The antibiotic-resistance rates for piperacillin/tazobactam and cefoperazone sulbactam were 13.16%, whereas for amoxicillin/clavulanic acid 18.42%. Only one carbapenem-and tigecycline-resistant *E. coli* strain was detected (Figure 5B).

A total of 36 ESBL-positive strains were detected, including 20 (20/36, 55.56%) in the *E. coli* group and 16 (16/36, 44.44%) in the *K. pneumonia* group. In addition, the number of ESBL-positive strains increased from 2018 (27.78%; 5/18) to 2021 (50.00%; 16/32). The antibiotic resistance of ESBL-positive strains was 33.33% for amoxicillin/clavulanic acid, 36.11% for cefoperazone sulbactam, 30.56% for piperacillin/tazobactam, 5.56% for imipenem, and 11.11% for tigecycline (Table 3).

**TABLE 3:** The antibiotic resistance of ESBL-positive strains.

Antibiotic name	Number	R + I%	95% CI	MIC50
Amoxicillin/Clavulanic acid	36	33.33	19.1–51.1	16
Cefoperazone/Sulbactam	36	36.11	21.3–53.8	16
Piperacillin/Tazobactam	36	30.56	16.9–48.3	16
Cefuroxime	35	97.14	83.4–99.9	64
Ceftazidime	32	59.38	40.8–75.8	32
Ceftriaxone	36	100	88.0–100	64
Cefepime	36	77.78	60.4–89.3	32
Cefoxitin	36	11.11	3.6–27.0	4
Ertapenem	36	2.78	0.1–16.2	0.125
Imipenem	36	5.56	1.0–20.0	0.25
Amikacin	35	8.57	2.2–24.2	2
Levofloxacin	36	58.33	40.9–74.0	8
Trimethoprim/Sulfamethoxazole	36	75	57.5–87.3	384
Tigecycline	36	11.11	3.6–27.0	0.5

A total of 25 strains of *A. baumannii* were detected, with most of them being multidrug-resistance (MDR) strains. The antibiotic-resistance rate of these strains was 90.48% for meropenem, 80.00% for ciprofloxacin, 85.71% for ticarcillin clavulanic acid, 84.00% for piperacillin/tazobactam, 56% for cefoperazone sulbactam, 86.36% for ceftazidime, 22.73% for minocycline, and 16.00% for amikacin. However, polymyxin-resistant strains were not detected (Figure 5C).

A total of 16 strains of *P. aeruginosa* were isolated, including three carbapenem-resistant strains. These strains exhibited an antibiotic-resistance rate of 100% for amoxicillin, 21.42% for clavulanic acid, 6.67% for cefepime, 7.14% for meropenem, 25.00% for piperacillin, tazobactam, and levofloxacin, and 6.25% for tobramycin. However, amikacin-, gentamicin-, and colistin-resistant strains were not observed (Figure 5D).

One strain (1/38, 2.63%) of carbapenem-resistant *E. coli* (CRE) and 10 strains (10/44, 22.73%) of carbapenem-resistant *K. pneumonia* (CRKP) were detected. Three strains (3/16, 18.75%) of *P. aeruginosa* were resistant to carbapenems (CRPA). One strain (1/38, 2.63%) of *E. coli*, seven strains (7/44, 15.91%) of *K. pneumonia*, and four strains (4/25, 16.00%) of *A. baumannii* were resistant to tigecycline.

#### Gram-Positive Bacteria

Among the Gram-positive bacteria, 21 strains of *S. aureus* were detected, including four methicillin-resistant *S. aureus* (MRSA) (4/21, 19.05%). Among the penicillin-resistant strains, the production rate of β-lactamase was 80.95%, the resistance rates for penicillin, vancomycin, and levofloxacin were 71.43, 4.76, and 9.52%, respectively (Figure 5E).

A total of 43 cases of enterococci were detected, including one vancomycin-resistant enterococci (VRE) strain. The overall antibiotic-resistant rates of the enterococcal strains were 81.40, 83.72, 95.23, and 2.32% for penicillin, levofloxacin, clindamycin, and vancomycin, respectively. However, tigecycline-and linezolid-resistant strains were not detected (Figure 5F).

#### Fungus

**FIGURE 6:**
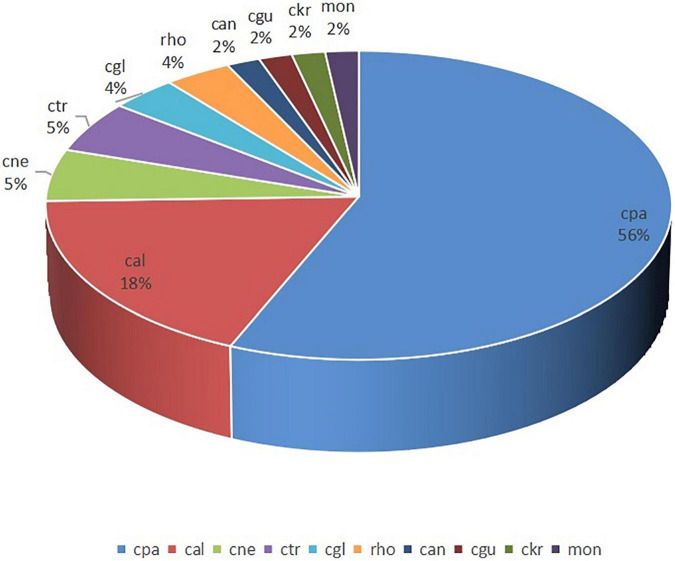
Distribution of fungal strains (%). cpa, *C. parapsilosis;* cal, *C. albicans;* cne, *Cryptococcus neoformans;* ctr, *C. tropicalis;* cgl, *C. glabrata;* rho, *Rhodotorula;* can, *Candida spp;* cgu, *Candida guilliermondii;* ckr, *C. krusei;* and mon, *Monilia.*

A total of 55 fungal strains were detected, including 31 *C. parapsilosis*, 10 *C. albicans*, 3 *Cryptococcus neoformans*, 3 *C. tropicalis*, and 8 other strains. Compared with the high incidence of *C. parapsilosis*, the occurrence of *C. albicans* was lower (Figure 6). The antibiotic-resistance rate of these strains for voriconazole, fluconazole, and itraconazole was 5.77%. However, amphotericin B-and flucytosine-resistant strains were not observed (Figure 5G).

## Discussion

According to the Hour-1 Bundle of SCC, antibiotic treatment should be initiated within 1 h, and etiological examination should be performed to treat critical patients with severe infection and septic shock (9). A delay in prescribing an adequate empiric antibiotic therapy may result in increased mortality, whereas the early prescription of effective antimicrobial treatment is linked to improved clinical outcomes (11–15). In the absence of blood culture reports, clinicians in ICU prescribe empiric broad-spectrum therapy with one or more intravenous antimicrobials to cover all likely pathogens (9). The use of broad-spectrum antibiotics is closely related to the emergence of bacterial resistance, with Gram-negative bacilli exhibiting the highest resistance. Intrinsic, adaptive, and acquired antimicrobial resistance led to the emergence of MDR, XDR, and PDR strains (16, 17). This study found that among the indexes related to the severity of the disease, patients with Gram-negative BSI used more vasopressor, exhibited the highest SOFA score, highest CR level, lowest GCS score, and lowest PLT count. Subgroup comparison showed Gram-negative group patients with lower mean blood pressure and higher ALT, AST, and TBIL values compared with Gram-positive group patients. Therefore, clinicians should pay more attention to infections caused by Gram-negative strains.

The β-lactamase producing Gram-negative bacteria threaten critical care of patients, as they often cause MDR infections. Major β-lactamase families include plasmid-mediated ESBLs, AmpC cephalosporinases, and carbapenemases (18). In this research period, ESBLs were the most common β-lactamases, while carbapenemases were not checked in our hospital.

Extended-spectrum β-lactamases are one of the most popular reasons for intrinsic resistance in bacteria. According to our report, 52.63% of *E. coli* were ESBL positive, which is similar to the rate in China (19) but higher than that in the United States (20). Apart from *E. coli*, 36.37% of *K. pneumonia* were ESBL positive (16/44, 36.36%). Increasing rates of ESBL-positive pathogens have been reported in many countries, leading to an increased focus on this group (21, 22). In our study, the ESBL positivity rate increased from 22.22% in the first half of 2018 to 52.38% in the second half of 2020 (Appendix 1); however, the difference was not statistically significant, which may be related to the limited data. Moreover, the carbapenems-resistance rate in *K. pneumonia* was 22.73%. According to the China Antimicrobial Surveillance Network (CHINET), the prevalence of meropenem-resistant *K. pneumonia* increased in China from 9.0% in 2011 to 26.3% in 2018 and fluctuated between 24.2–27.1% from 2019 to 2021. Carbapenem is the preferred treatment for infections outside of the urinary tract caused by ESBL-E (23). The link between carbapenem consumption and the emergence of carbapenem resistance has been indicated in various studies (24). Therefore, reducing the irrational use of carbapenem antibiotics is critical for healthcare systems. Mark D. Lesher et al. reported that removing the ESBL designation from microbiology reports could decrease the prescription of carbapenems from 48.4 to 16.1% and increase the use of β-lactam–β-lactamase inhibitor combinations from 19.4 to 61.3% (25). In fact, piperacillin/tazobactam is our center’s most widely used antibiotic (Appendix 2) for suspected Gram-negative bacterial infections. But the susceptibility of *E. coli*, *K. pneumonia*, and *P. aeruginosa* to piperacillin is lower (66/98, 67.35%) than that to carbapenems (82/98, 83.67%). The data of ESBL positive strains is few, but the increasing trend of ESBL-positive rate from 2018 (27.78%; 5/18) to 2021 (50.00%; 16/32) is observed. Whether the increasing trend is related to the high usage of piperacillin tazobactam remains to be further studied. According to a study by Patrick N A Harris et al. among patients with *E coli.* or *K. pneumoniae* BSI and ceftriaxone resistance, compared with meropenem treatment, definitive treatment with piperacillin-tazobactam did not result in non-inferior 30-day mortality (26). Stewart AG et al. reported that piperacillin-tazobactam might lead to more microbiological failures among patients with bloodstream infection due to AmpC producers (27). Piperacillin tazobactam is not suitable for empirical treatment of ESBL positive bloodstream infection. According to our research results, the drug resistance rate of tigecycline, polymyxin, and amikacin against Gram-negative bacteria is lower than that of piperacillin/tazobactam and carbapenem, which can be a choice for Gram-negative bacteria infection. However, the plasma concentration of tigecycline is low and is not recommended for patients with bloodstream infection. Aminoglycoside antibiotics and colistin have adverse reactions of nephrotoxicity. Patients with bloodstream infection are usually complicated with organ function injury. AKI is a common complication. So the application of aminoglycoside drugs and colistin in ICU critical patients is limited. Aminoglycoside antibiotics and colistin are more suitable for combination therapy (23).

In this report, the detection rate of Gram-positive strains, especially *S. epidermidis*, in blood culture was high, which is close to the detection rate reported by CHINET but lower than that of Diekema et al. (28). Coagulase-negative staphylococcus was a common colonization bacterium on the skin. However, partial coagulase-negative Staphylococcus strains were identified in the BSI patients and included in the statistical analysis during the retrospective analysis. *S. epidermidis* was the most detected, with 38 strains in 3 years and 13 discharged cases with contaminated strains. Twenty-five patients with positive *S. epidermidis* in blood culture were treated with vancomycin or linezolid. Most were critical patients with damaged skin barriers caused by skin damage or dermatic cellulitis, low immune function, and disposed to Gram-positive cocci infections. During the 3 years of this study, the use of vancomycin showed a gradual upward trend, and the possibility of antibiotic abuse could not be excluded. In our study, the Gram-positive BSI maintained a low resistance rate to vancomycin, indicating that vancomycin could be used as an empirical drug. However, the use of vancomycin against coagulase-negative staphylococcus should be restrained to reduce medical consumption and the emergence of antibiotic-resistant bacteria.

Risk factors for invasive candidiasis include the extensive use of invasive procedures and devices, broad-spectrum antimicrobial agents, advanced life support, and aggressive chemotherapy (29). In this study, the incidence of fungemia in ICU was 0.73% (55 patients/7,577 ICU admissions), which was higher than that of 0.32% reported by Guo F et al. (306 patients/96,060 ICU admissions) (30). Nearly two-thirds of fungemia cases were ICU-acquired infections. Moreover, the occurrence of *C. parapsilosis* was higher than that of *C. albicans*, which was following the results of Pfaller MA et al. who reported a decreased detection of *C. albicans* and increased isolation of *C. glabrata* and *C. parapsilosis* (31). Previous studies have indicated that patients requiring prolonged use of a central venous catheter or indwelling device are at an increased risk of *C. parapsilosis* infection (32). Therefore, it is suggested that antifungal treatment should be added to the treatment regime of high-risk patients in ICU. In this study, the overall resistance rate of fluconazole was 10.91%, indicating its use as an empirical antifungal drug. Because of the high incidence of *C. parapsilosis* and to reduce the cases of BSI caused by *C. parapsilosis*, catheter maintenance in clinical operations should be given importance. The high positivity rates of methicillin-resistant *S. epidermidis* and *C. parapsilosis* suggest that our hospital needs to strengthen further the prevention and control of ICU-acquired infections or catheter-related infections (33).

Many studies have employed next-generation sequencing (NGS) to reduce the waiting time for blood culture reports (34, 35). Moreover, rare microorganisms could be detected by NGS. However, because of the high expense, this technology could not be used in our hospital. In this study, the level of peripheral blood leukocytes, an indicator of inflammatory reaction, did not vary among the Gram-positive, Gram-negative, and fungal groups. The level of PCT in the Gram-negative group was significantly higher than that of the other two groups. Studies have confirmed that PCT, a common clinical monitoring index, can be used to distinguish infectious fever from non-infectious fever (36). Dynamically monitoring the changes in PCT levels could determine the time to initiate antibiotic treatment and discontinue the same, thereby reducing the duration of antibiotic use without affecting the prognosis (37–39). The plasma PCT levels of patients with Gram-negative bacterial infections were higher than that of the patients with Gram-positive and the plasma PCT levels were higher in bacterial BSI group than that in fungal BSI group, which was consistent with the results of earlier studies (40, 41). However, it was not an effective indicator to distinguish, thereby indicating the need for further large-scale research. Although no differences in PCT concentrations were observed among the cases with *E. coli, K. pneumonia, P. aeruginosa*, and *A. baumannii* infections, blood culture reports revealed two or three times higher PCT values in *E. coli* and *P. aeruginosa* infections compared with that of *K. pneumonia and A. baumannii* infections. Daniel O. Thomas-Rüddel et al. reported that Streptococci, *E. coli*, and other Enterobacteriaceae detected in blood culture were associated with three times higher PCT values in a linear regression model (40). Although many studies have demonstrated the advantages of PCT in the diagnosis and treatment of BSI, especially Gram-negative BSI, more experimental data and/or more inflammatory indicators are needed for the differential diagnosis of infectious pathogens.

## Conclusion

Among the bloodstream infection strains in ICU, Gram-negative bacteria have the highest drug resistance rate, and will cause more serious brain damage, renal function damage and thrombocytopenia. So clinician should pay more attention to the treatment of Gram-negative bacteria in patients with bloodstream infection in ICU. The test index of PCT can be used to distinguish Gram-negative bacteremia from Gram-positive and bacteremia from fungemia but not as an effective indicator, thereby indicating the need for further large-scale research. This study has the following limitations: (1) since the included cases were single-center samples, the findings of this study cannot be generalized to other regions; (2) this study is prone to bias because of its retrospective characteristic; (3) because of the small sample size, the research results and conclusions are only for reference; and (4) due to the limited conditions, the carbapenem-resistance gene was not detected.

## Data Availability Statement

The original contributions presented in the study are included in the article/supplementary material, further inquiries can be directed to the corresponding authors.

## Ethics Statement

The studies involving human participants were reviewed and approved by the Ethics Committee of Tianjin Medical University General Hospital, China. The ethics committee waived the requirement of written informed consent for participation. Written informed consent was not obtained from the individual(s) for the publication of any potentially identifiable images or data included in this article.

## Author Contributions

H-NW, E-YY, and MP contributed to the conception and design of the study. H-NW organized the database and wrote the first draft of the manuscript. W-BL and E-YY performed the statistical analysis. Q-YZ, K-lX, H-NW, and MP wrote sections of the manuscript. All authors contributed to manuscript revision, read, and approved the submitted version.

## Conflict of Interest

The authors declare that the research was conducted in the absence of any commercial or financial relationships that could be construed as a potential conflict of interest.

## Publisher’s Note

All claims expressed in this article are solely those of the authors and do not necessarily represent those of their affiliated organizations, or those of the publisher, the editors and the reviewers. Any product that may be evaluated in this article, or claim that may be made by its manufacturer, is not guaranteed or endorsed by the publisher.

## Appendix

**Appendix 1:** ESBL-positivity rate in the first half of the years 2018–2020.

	2018 First half	2018 second half	2019 First half	2019 second half	2020 First half	2020 second half	*P*-value
ESBL positivity rate (%)	22.22	33.33	38.46	50.00	45.45	52.38	0.143

**Appendix 2:** Antibiotic consumption in the first half of the years 2018–2020.

Antibiotic consumption	2018 First half	2018 second half	2019 First half	2019 second half	2020 First half	2020 second half	*P*-value
Piperacillin/tazobactam	1300.82	1425.86	1721.89	1491.75	1381.18	1536.43	0.566
Cefuroxime	218.50	369.25	622.75	675.00	703	611.5	**0.041**
Cefotaxime sodium	82.125	34.625	114.25	62.375	48.625	157.25	0.397
Cefepime	769	739.5	1224.5	1425.5	1376	1023.5	0.201
Cefoperazone Sulbactam Sodium	546	622.125	820.875	937.125	657	696.375	0.477
Cefatriaxone	136	185	194	286.5	147.5	282.5	0.223
Cefazolin	62.67	65.00	96	167	101.33	178.33	**0.049**
Cefoxitin	271.83	227.67	391.67	371.83	196	350.33	0.729
Levofloxacin	58	71	102	145	102	144	**0.037**
Imipenem cilastatin	159	232.5	573.5	435	244	295.5	0.701
Meropenem	103.5	117.75	284.5	123.75	64.5	57.75	0.489
Tigecycline	193.5	177.5	564	352.5	304.5	205.5	0.875
Linezolid	151.83	226.33	509.83	436.67	287.33	207.17	0.781
Vancomycin	60.25	77.75	85.25	116.25	105	129.5	**0.004**
Fluconazole	192	154	293	161	202	240	0.622
Caspofungin	3	13	16	14	7	84	0.139
Voriconazole	31.5	83	170.5	151.5	30	205.5	0.317
Ceftazidime	67.75	55.5	78	120.25	177.25	221.5	**0.005**
Total	4407.275	4877.36	7862.515	7473	6134.215	6626.635	0.247

*Antibiotic consumption was expressed in defined daily dose(DDD) in every six months. The rising consumption was detected in Cefuroxime, Cefazolin, Levofloxacin, Vancomycin and Ceftazidime in three years.*
