# Corrigendum: Microbiological and clinical characteristics of bloodstream infections in general intensive care unit: A retrospective study

**DOI:** 10.3389/fmed.2023.1174935

**Published:** 2023-03-21

**Authors:** He-Ning Wu, Er-Yan Yuan, Wen-Bin Li, Min Peng, Qing-Yu Zhang, Ke-liang Xie

**Affiliations:** ^1^Department of Critical Care Medicine, Tianjin Medical University General Hospital, Tianjin, China; ^2^Department of Geriatrics, Tianjin Medical University General Hospital, Tianjin, China; ^3^Department of Gastroenterology, Tianjin Medical University General Hospital, Tianjin, China; ^4^Department of Anesthesiology, Tianjin Institute of Anesthesiology, Tianjin Medical University General Hospital, Tianjin, China

**Keywords:** bloodstream infection, antibiotic resistance, procalcitonin, multidrug-resistance, vancomycin-resistant enterococci (VRE)

In the published article, there was an error in the author list, and author He-Ning Wu and Er-Yan Yuan were erroneously excluded. The corrected author list appears below.

He-Ning Wu^1^^†^, Er-Yan Yuan^1^^†^, Wen-Bin Li^1,2^, Min Peng^1^, Qing-Yu Zhang^1,3*^ and Ke-liang Xie^1,4*^

^†^These authors have contributed equally to this work

The authors apologize for this error and state that this does not change the scientific conclusions of the article in any way. The original article has been updated.

